# Arteriobiliary Fistula and Post-traumatic Pseudoaneurysm in the Right Hepatic Artery

**DOI:** 10.7759/cureus.100269

**Published:** 2025-12-28

**Authors:** Ketzarly G Gallardo Durán, Adrian E Aranda Chavez, Rodolfo J Castelló Magallanes

**Affiliations:** 1 Digestive and Endocrine Surgery, Instituto Mexicano del Seguro Social, Torreon, MEX; 2 Surgical Oncology, Instituto Mexicano del Seguro Social, Torreon, MEX

**Keywords:** biliary injury, fistula arteriobiliar, haemobilia, laparoscopic cholecistectomy, right hepatic artery (rha)

## Abstract

Hemobilia is a rare but potentially life-threatening cause of upper gastrointestinal bleeding, defined as hemorrhage into the biliary tree resulting from an abnormal communication between the hepatic vasculature and the biliary system. It is most commonly associated with hepatobiliary surgical interventions and vascular injuries. We report the case of a female in her 20s with no relevant medical history, who underwent an apparently uncomplicated laparoscopic cholecystectomy in September 2023 and subsequently presented in February 2024 with recurrent, intermittent episodes of hematemesis and hematochezia. Upper gastrointestinal endoscopy demonstrated active hemobilia. Contrast-enhanced computed tomography revealed intrahepatic bile duct dilatation, pneumobilia, and a right hepatic artery pseudoaneurysm, establishing the diagnosis. The patient rapidly deteriorated, developing hemorrhagic shock and severe anemia, needing intensive care admission, endotracheal intubation, blood transfusion, and hemodynamic support. Definitive management was achieved through endovascular intervention with a stent, which remains the treatment of choice. This case highlights that hemobilia may present months after a cholecystectomy, often with intermittent or subtle bleeding, underscoring the importance of maintaining a high index of suspicion and recognizing this rare but serious complication early to prevent morbidity and mortality.

## Introduction

Hemobilia, defined as bleeding into the biliary tree, is a rare but potentially life-threatening condition first characterized by Sandblom in 1948 [1]. This condition typically results from an abnormal communication between the hepatic arterial system and the bile ducts, most commonly through arteriobiliary fistulae or pseudoaneurysms. The classic presentation, Quincke's triad of right upper quadrant pain, jaundice, and gastrointestinal bleeding, was originally described in 1871 and manifests in fewer than 40% of cases, often delaying diagnosis [2,3].

In recent years, iatrogenic injuries have emerged as the leading cause of hemobilia, driven by the increasing prevalence of hepatobiliary procedures such as laparoscopic cholecystectomy, liver biopsy, and percutaneous biliary interventions [4]. Laparoscopic cholecystectomy, although widely regarded as safe, carries a risk of vascular and biliary injuries, which may lead to complications including hepatic artery pseudoaneurysms or arteriobiliary fistulae [5,6]. Such injuries can result in arterial wall weakening, pathological hemodynamic changes, and eventual communication with the biliary tree, as documented in several case reports and case series [7-9].

Advancements in diagnostic imaging have significantly improved the recognition of hemobilia. Non-invasive modalities such as contrast-enhanced computed tomography (CT), Doppler ultrasonography, and magnetic resonance imaging (MRI) are instrumental in detecting pseudoaneurysms, active bleeding, and biliary obstruction [8,10]. However, angiography remains the diagnostic gold standard, offering both precise localization and immediate therapeutic capability [11,12].

The primary treatment for hepatic artery pseudoaneurysms and arteriobiliary fistulae is transcatheter arterial embolization (TAE), which demonstrates high success rates and a markedly lower morbidity profile compared with open surgery [6]. In selected cases, particularly when arterial preservation is desired, covered stent placement provides an effective alternative [11]. Esophagogastroduodenoscopy (EGD) and endoscopic retrograde cholangiopancreatography (ERCP) continue to play a complementary role, particularly in addressing concurrent gastrointestinal bleeding or biliary obstruction [13].

This report presents a case of hemobilia in a young female following laparoscopic cholecystectomy, presenting with hematemesis and melena. Imaging revealed a right hepatic artery aneurysm with associated arteriobiliary fistula. Successful management was achieved via endovascular stent placement, resulting in complete hemostasis and resolution of the fistula. This case highlights the importance of safe surgical practices, early recognition of hemobilia, and the expanding role of modern imaging and endovascular therapies in optimizing patient outcomes.

This case was previously presented as an ePoster at the 2025 SAGES Annual Meeting in Long Beach, California, USA (March 12-15, 2025).

## Case presentation

A 21-year-old female, with no significant past medical history, presented with intermittent episodes of hematemesis and hematochezia starting in February 2024. She had undergone a laparoscopic cholecystectomy in September 2023 due to cholelithiasis without apparent complications. 

The patient was referred to outpatient gastroenterology services for evaluation and underwent the following diagnostic studies. 

**Figure 1:**
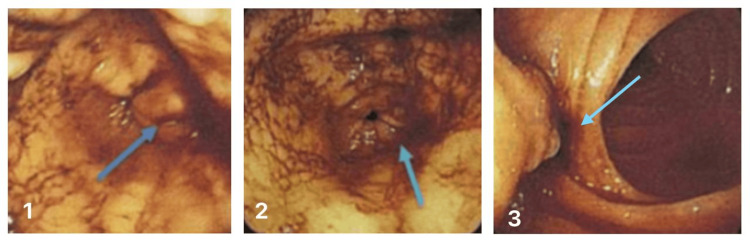
Esophagogastroduodenoscopy (EGD) showing bleeding from the major papilla Duodenum, the second portion, active oozing bleeding *(*image 1, blue arrow) and an adherent clot on the major papilla were noted.

Esophagogastroduodenoscopy (EGD) findings revealed chronic gastropathy with erosive changes in the gastric body and follicular gastritis in the antrum. The patient was diagnosed with upper gastrointestinal bleeding originating from the major papilla (Figure 1, images 2-3). The possibility of a stone passing through the papilla, causing tearing and subsequent bleeding, could not be ruled out. She went home with peptic disease medical management.

The second EGD two weeks later showed large amounts of blood clots in the stomach, which were aspirated (500 mL). Active oozing from the mucosa (Figure 1) of the second portion of the duodenum was observed, with an adherent clot at the major papilla. Hemostasis was achieved with 4 mL of 1:1000 adrenaline injection.

Three days later, a new EGD showed a 10-mm subepithelial lesion in the gastric antrum.

**Figure 2:**
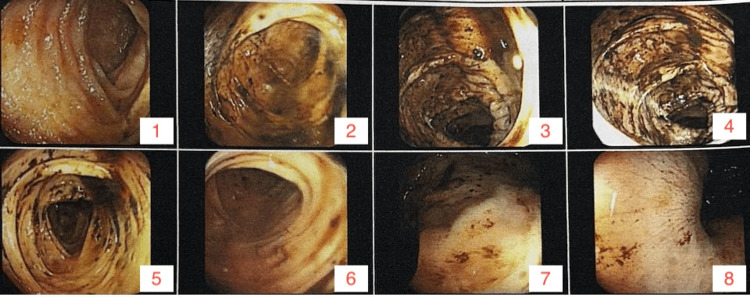
Colonoscopy showing evidence of upper gastrointestinal bleeding Ileum (images 1-2), colon (images 3-5), and rectum (images 6-8) were without macroscopic abnormalities.

**Figure 3:**
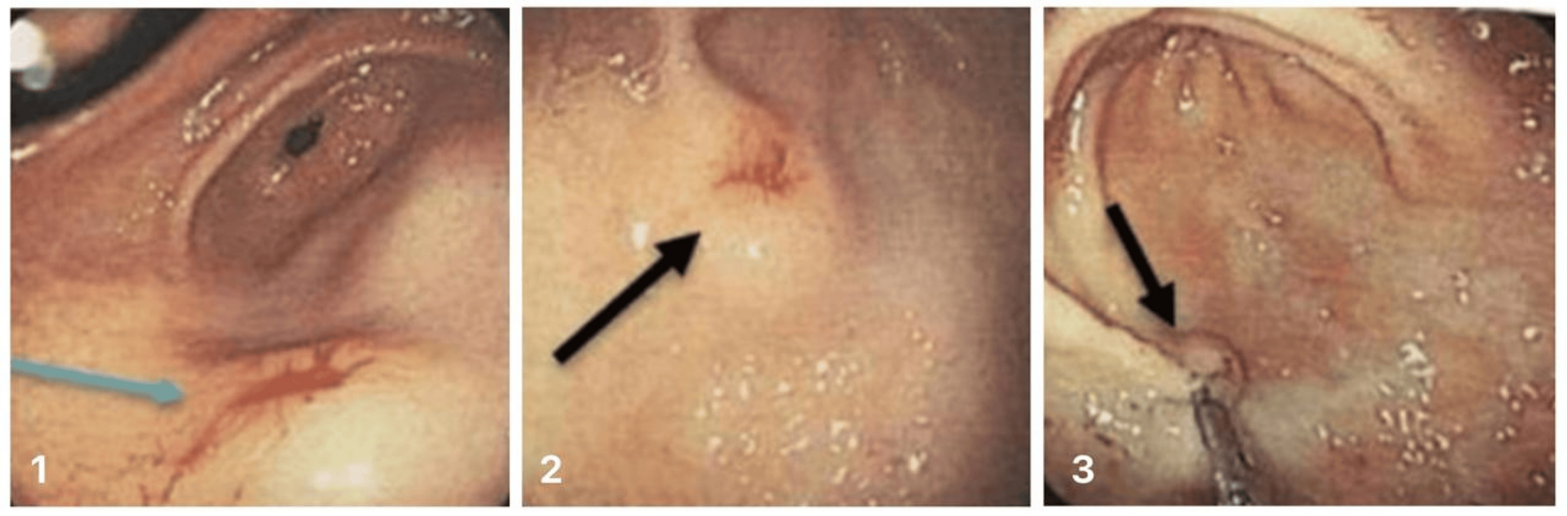
Subsequent esophagogastroduodenoscopy (EGD) An 18 mm subepithelial lesion (image 1, blue arrow) in the antrum with a 6 mm central ulcer (Forrest IIC) showing minimal bleeding (images 2-3, black arrows) was treated with adrenaline infiltration and hemoclip placement.

After a colonoscopy was performed, melena was observed in the intestinal lumen, with no other abnormalities (Figure 2). Afterward, another EGD (Figure 3) was performed 48 hours later, revealing hyperemic and pale areas alternating in the mucosa of the gastric body and antrum, with a fine nodular pattern. An 18-mm subepithelial lesion in the antrum with a 6-mm mucosal defect was noted, exhibiting scant bleeding upon manipulation. Hemostasis was achieved with a 1:20,000 adrenaline injection and placement of a hemoclip. The duodenal papilla showed bile outflow without evidence of bleeding. Diagnosis of chronic gastropathy was made without further procedures, only outpatient follow-up. 

Five days later, the patient was admitted under the care of the gastroenterology service due to signs of severe anemia and no improvement in symptoms. Two days after the hospitalization, a new EGD was performed due to the persistent signs of constant bleeding and refractory anaemia, finding only self-limiting oozing from the mucosa at the gastroesophageal junction, with probable but not active hemobilia. 

**Figure 4:**
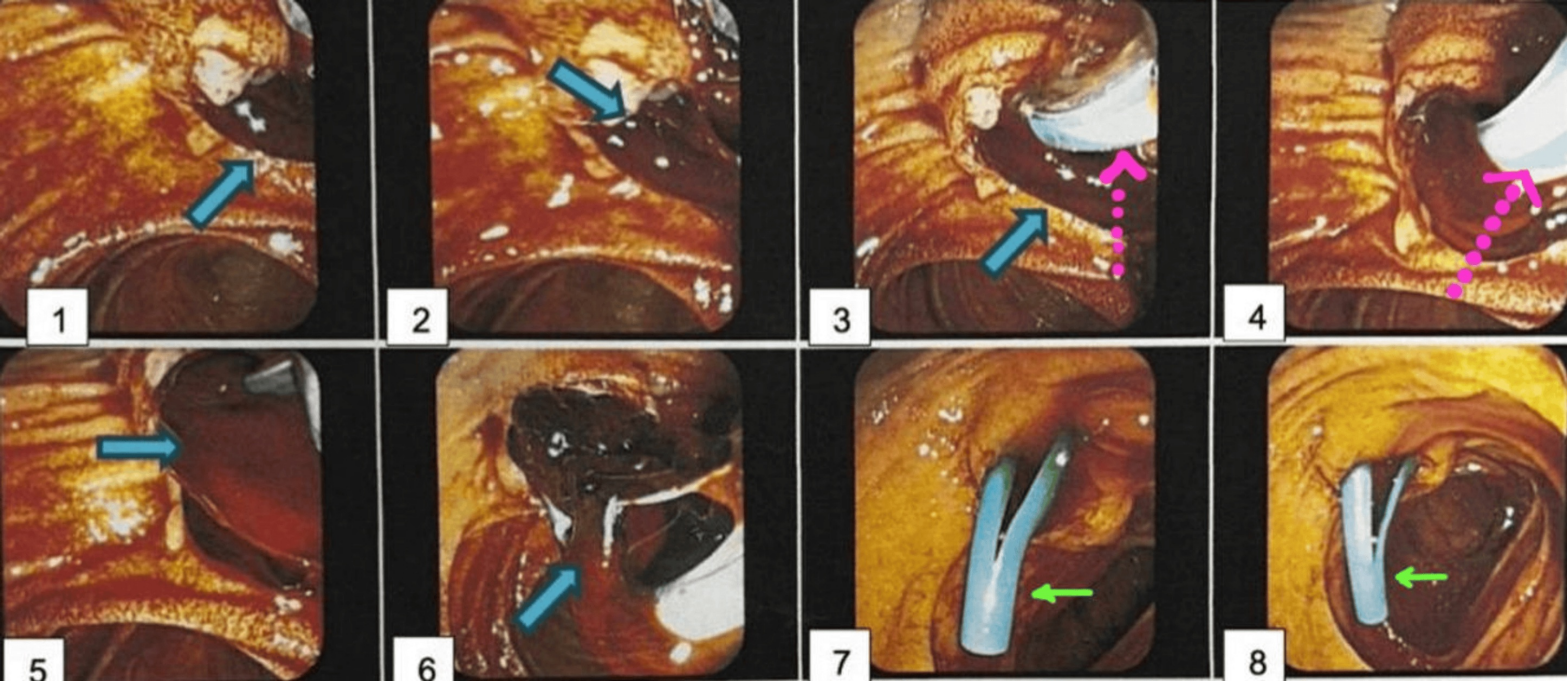
Endoscopic retrograde cholangiopancreatography (ERCP) indicating hemobilia of undetermined etiology Images 1-3: the ampulla of Vater swollen and with blood clots (blue arrows) Images 3-4: sphinteroctome and wire for selective biliary tract cannulation (pointed pink arrow) Images 5-6: abundant fresh blood and clots during cannulation Images 7-8: 10 Fr (7 cm) biliary stent (green arrow) was placed; fresh blood and bile drained

**Figure 5:**
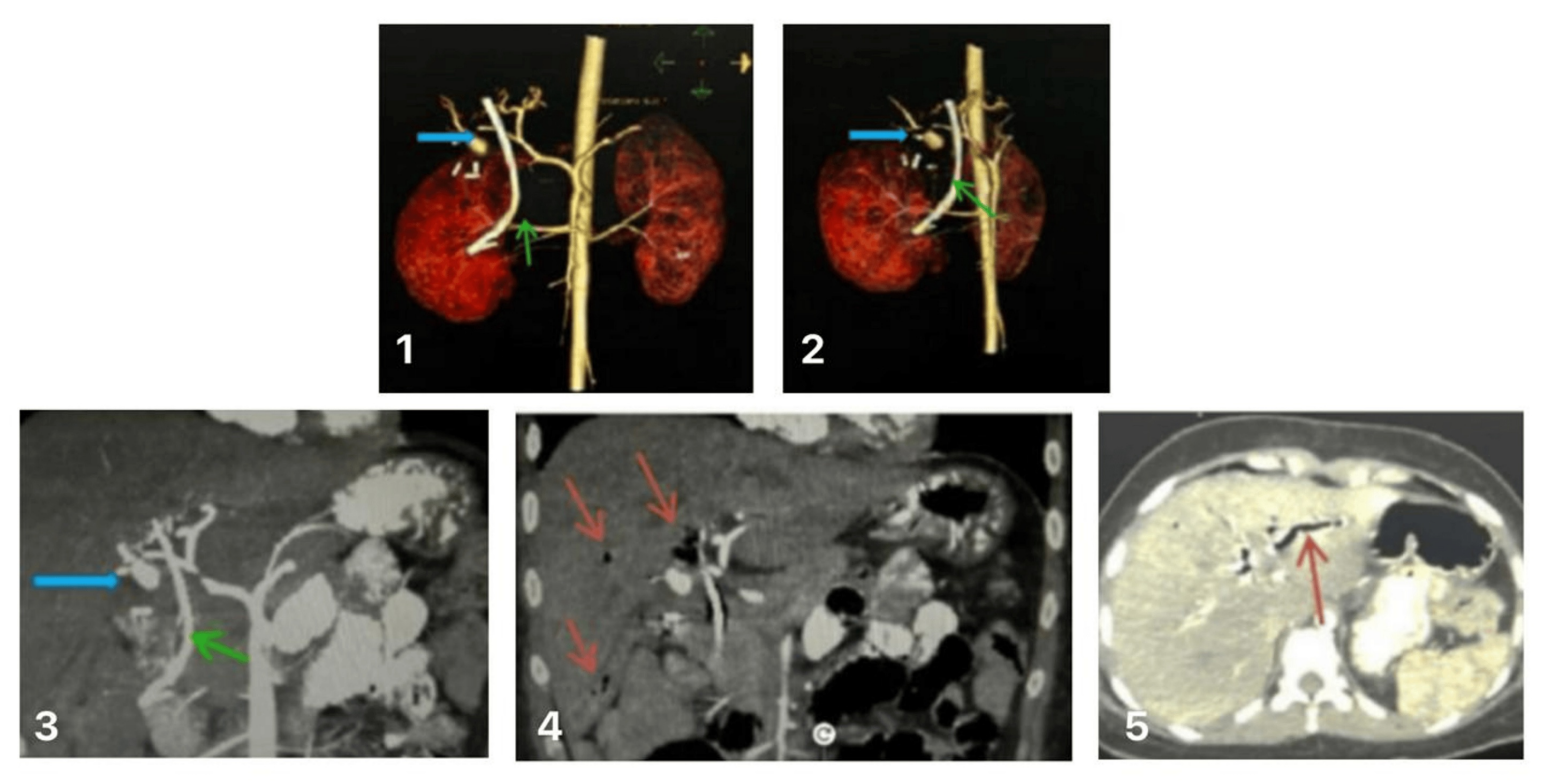
Computed tomography angiography (CTA) with 3D vascular reconstruction consistent with a pseudoaneurysm of the right hepatic artery branch Images 1-2: hepatic artery pseudoaneurysm (blue arrow); rounded, well-defined structure (11x14x15 mm) with a small filiform pedicle connected to the right hepatic artery, 20 mm from its origin Image 3: biliary stent (green arrow); proximal end in the common hepatic duct, distal end in the ampullary region of the common bile duct Images 4-5: biliary dilation and pneumobilia (red arrows) are likely due to recent instrumentation (endoscopic Retrograde cholangiopancreatography) and surgery (laparoscopic cholecystectomy)

The day after, the patient developed severe hemorrhagic shock and was admitted to the ICU, and a general surgery consultation was requested. An ERCP (Figure 4) on the same day of admission to the ICU showed active hemobilia of undetermined etiology. The biliary tract was selectively cannulated using a sphincterotome and a guidewire. Surgeons requested an abdominal CTA scan, which revealed intrahepatic biliary dilation and pneumobilia consistent with recent instrumentation (ERCP) and surgery (laparoscopic cholecystectomy). A well-defined, rounded lesion measuring 11×14×15 mm with a small pedicle connected to the right hepatic artery 20 mm from its origin was identified (Figure 5). The lesion demonstrated vascular behaviour consistent with a right hepatic artery pseudoaneurysm. 

Upon initial evaluation by the general surgery department, following initial hemodynamic resuscitation, the patient was intubated and sedated. Clinical findings included previous evidence of volume depletion, with a semi-globose, soft, and depressible abdomen, with no evidence of acute abdomen; an orogastric tube with 10 mL of bilious output; a urinary catheter with a urine output index of 1.9 mL/kg/h; weak peripheral pulses; and a capillary refill time of 3 seconds. At the time of assessment, vital signs were blood pressure 106/48 mmHg, heart rate 90 bpm, respiratory rate 18 breaths/min, temperature 37.4°C, and oxygen saturation 100% on 25% FiO₂, consistent with a post-resuscitation state. Laboratory findings are summarized in Table 1.

**Table 1:** Laboratory findings in the ICU

Test	Result	Reference range
Hemoglobin	5.5 g/dL	Male: 13.5-17.5 g/dL; female: 12.0-15.5 g/dL
Hematocrit	16.40%	Male: 41-53%; female: 36-46%
Leukocytes	7.69 ×10³/µL	4.0-11.0 ×10³/µL
Platelets	300 ×10³/µL	150-400 ×10³/µL
Prothrombin time (PT)	17.6 s	11-14 s
PTT (activated partial thromboplastin time, aPTT)	35.2 s	25-35 s
International normalized ratio (INR)	1.55	0.8-1.2
Creatinine	0.5 mg/dL	Male: 0.7-1.3 mg/dL; female: 0.6-1.1 mg/dL
Sodium	134 mEq/L	135-145 mEq/L
Potassium	3.8 mEq/L	3.5-5.0 mEq/L
Total bilirubin	2 mg/dL	0.1-1.2 mg/dL
Direct bilirubin	0.1 mg/dL	0.0-0.3 mg/dL
Indirect bilirubin	1.9 mg/dL	Calculated (total – direct)
Lactate dehydrogenase (LDH)	164 U/L	140-280 U/L
Alkaline phosphatase (ALP)	195 U/L	44-147 U/L
Aspartate aminotransferase (AST)	107 U/L	10-40 U/L
Alanine aminotransferase (ALT)	130 U/L	7-56 U/L

**Figure 6:**
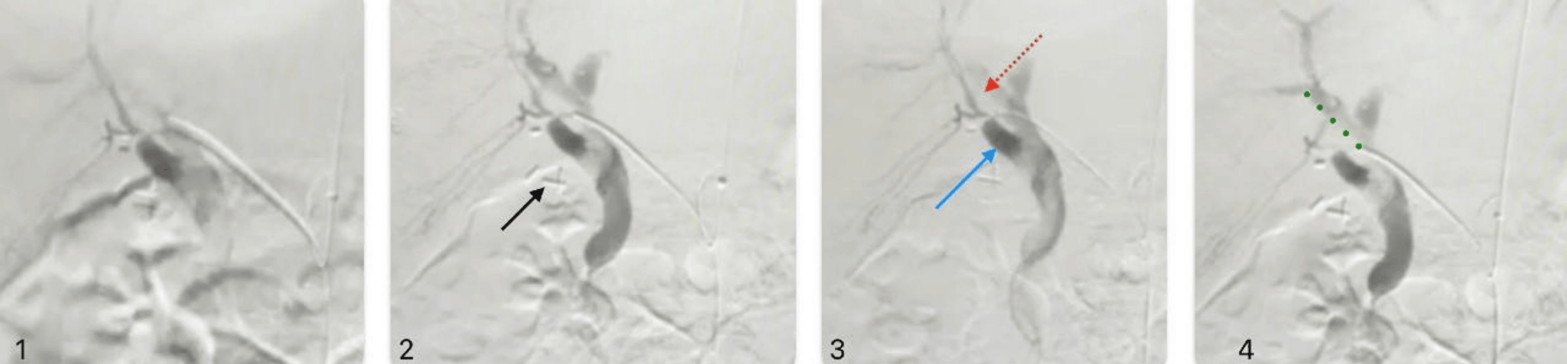
Arteriography revealing an arteriobiliary fistula Image 1: cannulated common hepatic artery Image 2: clips of the previous cholecystectomy Images 3-4: arterio-biliary fistula (blue arrow) with communication between the right hepatic artery (red dotted arrow) and the right hepatic bile duct (green dots)

On the same day, diagnostic arteriography revealed an arteriobiliary fistula (Figure 6), with communication between the right hepatic artery and the right hepatic bile duct.

**Figure 7:**
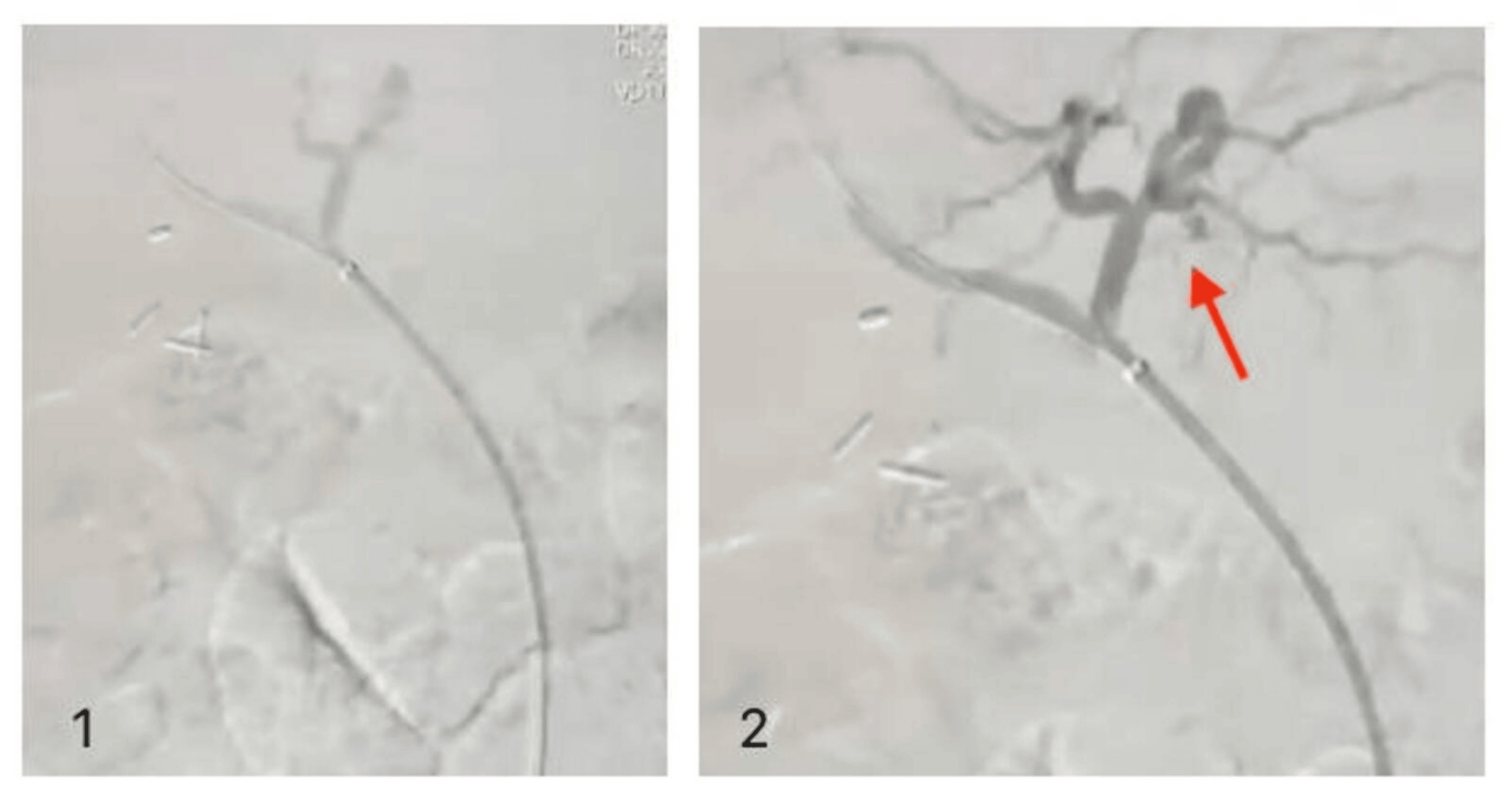
Post-procedure arteriography showing no evidence of leakage A covered Bentley 2.5×18 mm balloon-mounted stent was placed in the right hepatic artery. Image 1: cannulated artery Image 2:adequate blood flow (red arrow)

A covered Bentley 2.5×18 mm balloon-mounted stent was placed in the right hepatic artery. Post-procedure arteriography confirmed no extravasation of contrast (Figure 7). The patient was admitted to the ICU.

The patient demonstrated significant clinical improvement, with stabilization of hemodynamic parameters and resolution of bleeding. Post-treatment follow-up, including outpatient clinic visits and imaging studies, confirmed the absence of pancreatitis, stent thrombosis, recurrent bleeding, stent-related complications, or new vascular abnormalities.

She was discharged home four days later, with outpatient follow-up, maintaining good clinical progress without complications.

These favourable outcomes highlight the importance of multidisciplinary care involving gastroenterology, interventional radiology, and surgery.

## Discussion

Hemobilia, defined as bleeding into the biliary tract from a communication between the biliary system and adjacent blood vessels, represents a rare but potentially life-threatening cause of upper gastrointestinal hemorrhage [5]. Although once predominantly attributed to trauma, the widespread adoption of hepatobiliary interventions and laparoscopic cholecystectomy has shifted the etiology toward iatrogenic sources [6]. In the post-laparoscopic era, vascular complications-particularly involving the right hepatic artery or its branches-constitute an important subset that often eludes early recognition [7].

During laparoscopic cholecystectomy, the proximity of the cystic duct and cystic artery to the common hepatic duct and right hepatic artery makes these structures vulnerable to thermal or mechanical injury [8]. Such an insult may initially appear clinically silent but can induce gradual arterial wall weakening, resulting in pseudoaneurysm formation. Over time, pulsatile forces and local inflammation may promote rupture into the biliary tree, creating an arteriobiliary fistula [9]. Similar mechanisms have been reported in multiple case series describing delayed-onset hemobilia due to pseudoaneurysms of the right hepatic artery or cystic artery stump after technically challenging cholecystectomies [10]. This delayed evolution, ranging from weeks to months, explains why postoperative hemobilia is frequently misattributed to mucosal or gastric sources, as occurred in our case, where endoscopy initially revealed gastric erosion.

In Mexico and much of Latin America, the adoption and widespread training in laparoscopic cholecystectomy occurred later than in high-income countries. As a result, the incidence of bile duct injury (BDI) remains higher than international benchmarks, with reported national rates ranging between 0.4% and 1.2%, compared with 0.1-0.5% in experienced centers worldwide [11]. This regional context is clinically relevant: biliary injuries often coexist with vascular injuries, particularly involving the right hepatic artery, due to the close anatomic relationship within the hepatocystic triangle. Vascular injuries, even when not immediately recognized, may predispose patients to delayed complications such as pseudoaneurysm formation and hemobilia. In regions where early postoperative follow-up is inconsistent and where diagnostic suspicion for such delayed vascular sequelae may be low, the clinical course can evolve insidiously, contributing to prolonged morbidity and underdiagnosis.

Current interventional guidelines advocate transcatheter arterial embolization (TAE) as the gold-standard treatment for vascular hemobilia, achieving hemostasis in up to 90% of cases [6]. Several case reports support the efficacy of coil embolization or covered stent placement in pseudoaneurysms of the right hepatic artery, emphasizing their role in preserving hepatic perfusion while achieving bleeding control [12]. In selected patients, particularly those with solitary pseudoaneurysms of major hepatic arteries, covered stent placement may be preferred to maintain hepatic arterial flow [13]. Surgical exploration is now reserved for refractory bleeding, failed embolization, or associated biliary injury requiring reconstruction. The 2022 European Society of Gastrointestinal Endoscopy (ESGE) guidelines emphasize a multidisciplinary management strategy involving interventional radiology, gastroenterology, and hepatobiliary surgery to optimize outcomes.

This case underscores the diagnostic challenges of delayed hemobilia following laparoscopic cholecystectomy, particularly in regions where technical variability in training and postoperative surveillance may increase the risk of overlooked vascular injuries. Recognition of its potential for late, subtle, or misleading presentations is essential to avoid prolonged morbidity. By highlighting the mechanisms, evolving etiologies, and current evidence-based management strategies, this report reinforces the importance of maintaining a high index of suspicion in post-cholecystectomy patients with unexplained anemia, recurrent upper gastrointestinal bleeding, or biliary obstruction [14]. Ultimately, timely diagnosis and multidisciplinary intervention remain critical to preventing life-threatening complications and improving clinical outcomes.

## Conclusions

Hemobilia remains a rare but important cause of upper gastrointestinal bleeding and must be considered in patients with recent hepatobiliary interventions. Although Quincke's triad, right upper quadrant pain, jaundice, and gastrointestinal bleeding, can offer a valuable diagnostic clue, its incomplete presentation frequently complicates early recognition. This case also underscores that hemobilia can develop long after an apparently uncomplicated laparoscopic cholecystectomy, and that early bleeding episodes may be subtle, transient, or misattributed to more common gastrointestinal disorders.

Endovascular management, including selective embolization and covered stent placement, provides highly effective, minimally invasive treatment for vascular complications associated with hemobilia. The favorable outcome achieved through covered stent deployment in this case illustrates the therapeutic potential of these techniques in restoring vascular integrity while preserving biliary function. Ultimately, the timely integration of multimodal imaging, particularly contrast-enhanced CT and angiography, with coordinated multidisciplinary intervention remains essential to prevent life-threatening complications and improve outcomes in patients with suspected hemobilia.
